# Physiology of the Kidney1Read before the Ontario Veterinary College Medical Society.

**Published:** 1895-05

**Authors:** H. Busman

**Affiliations:** Student


### PHYSIOLOGY OF THE KIDNEY.[1]

BY H. BUSMAN, STUDENT.

Review of Anatomy.—*Compound tubular glands.* Uriniferous tubes, bloodvessels, nerves, connective tissue, fibrous capsule. Sectionally, an external or cortical portion; internal or medullary. Cortical portion, dark reddish brown in color. Medullary, paler in color, denser, fibrous in appearance. Cortical portion contains Malpighian bodies, consisting of capillary loops, covered by Bowman's capsule; primary and secondary convoluted tubes; primary form, the loop of Henle; secondary, the tubes of Bellini.

*Blood supply.* Renal arteries, interlobular arteries, vasa afferentia, vasa efferentia, glomerulus, vasa recta. The three different sets of capillary vessels control the pressure and rapidity of the flow of blood in these organs as follows:

1. The capillaries in the glomeruli are loops collected into a tuft by their covering of epithelium. On account of (*a*) the afferent artery ending abruptly in the capillaries, and of the efferent vessel (*b*) being smaller than the afferent, and (*c*) breaking up into a secondary plexus of capillaries, the pressure and velocity within the glomerulus must be great.

2. The secondary capillary plexus investing the tubules can only be under comparatively trifling and less variable pressure (*a*) on account of the blood having first to pass through the capillaries of the glomerulus, (*b*) their sectional area is enormous when compared with that of the glomerular vessels, and, therefore, the flow of blood must be slow in proportion.

3. The straight vessels have long-meshed capillaries, which lie between the looped and straight tubules. In these straight vessels the blood flows with greater rapidity than in those around the convoluted tubules. The pressure of their blood, though less than that in the glomeruli, is greater than that in the interlobular capillaries.

The nerves of the kidneys consist of a number of small branches running along the renal artery and coming from the solar plexus, which is formed by the splanchnics and branches from the superior cord of the vagus. That the whole of the

[1] Read before the Ontario Veterinary College Medical Society.

nerves do not come from the splanchnics is proven by stimulation of a sensory nerve of the medulla oblongata, or of the spinal cord in the neck; will cause contraction of the renal vessels after both splanchnics have been cut, and section of the splanchnics does not always cause the renal vessels to dilate.

The nervous centre for the renal artery is like the chief vasomotor centre for the body generally in the medulla oblongata; some say that there are also subsidiary centres in the spinal cord and in the solar and mesenteric plexuses.

There are four centres in the medulla—a vaso-constrictor, vaso-dilator, secretory and inhibitory-secretory.

FUNCTIONS OF THE KIDNEY.—It has to get rid of all of the impurities of the blood, whether the result of normal or pathological change; it eliminates all injurious, useless or superfluous matter. We may see, then, that the kidney has a threefold action:

1. They remove from the body excess of water.
2. They excrete waste products.
3. An arrangement for the retention of water in the body by its reabsorption after it has washed out the waste products.

These functions are performed by three separate portions of the kidney:

1. The Malpighian corpuscles by a process of *osmosis* eliminate water along with some solids.
2. The convoluted tubules with epithelial cells are, in all probability, the chief structures for excreting waste products.
3. Usually one or more constrictions in the tubule serve for the purpose of preventing too rapid exit of the water, and thus allow time for its reabsorption in cases where its retention is desirable.

The process of secretion in the kidney was regarded by Bowman as consisting of the filtration of water from the vessels of the glomeruli into the tubules and the excretion of waste products by the epithelium lining the tubules. Ludwig, however, came to look upon it rather as a process of filtration and reabsorption; according to him a dilute solution of urea and salt was poured out from the Malpighian corpuscles and gradually concentrated by the absorption of water in its passage along the tubules. This theory had so many facts in its favor that it was for a long time exclusively adopted; but, latterly, Heidenhain, in an admirable series of experiments, has shown that such substances as *indigogen* are certainly excreted by the epithelium of the tubules. At the same time Hufner has shown by a comparison of the kidney in fishes, frogs, tortoises, birds and mammals that the form of the tubule closely agrees with that required for the reabsorption of water in each case.

Fishes, living as they do in water, do not require any apparatus for its retention in the body; in them, therefore, the tubule is short and wide, without any constriction to retard the outflow of fluid. In frogs we must have provision for the retention of water in the body, as in them evaporation takes place freely from the skin; in them we find, as might be expected, the tubule, and especially the contracted portion, very long. In tortoises, where no evaporation can take place, the tubule is short.

This, then, shows that the ideas advanced by Bowman and supported by Heidenhain are in the main true; the reabsorption of water, on which Ludwig lays so much stress, is also an important factor in the secretion of urine under different circumstances.

*Circumstances modifying the secretion of urine.* The amount of urine secreted depends very much on the difference of pressure in the Malpighian corpuscles and the tubules, for if the ureter be tied the pressure of urine in the tubule is increased, so that secretion is diminished, or even arrested, though the blood-pressure in the artery is great. A similar effect is produced by ligaturing the renal vein, for the blood accumulating in the venous plexus surrounds and compresses the tubules, and so prevents the flow of urine through them. A similar condition may occur from cardiac or pulmonary diseases that cause venous obstruction. But, unless under these exceptional circumstances, which alter the pressure in the tubules, the rapidity of the secretion of urine depends upon three factors:

1. Arterial pressure in the glomeruli. 2. The composition of the blood. 3. Agents acting on the secretory centres.

The pressure in the glomeruli may be raised by: 1. Increased arterial tension generally, which may be brought about by greater action of the heart, or by contraction of the bloodvessels in other vascular areas; by nervous stimulation, exposure to heat or cold, or the action of drugs. 2. By increased tension locally, which may occur by dilatation of the renal arteries, *e. g.*, from secretion of the vaso-constrictor nerves or stimulation of the vaso-dilators.

The pressure of blood in the glomeruli may be diminished generally: 1. By failure of the heart's action. 2. By dilatations of the vessels of large areas, as the intestines, muscles or skin, which may be caused by exposure to warmth, by the action of drugs or by paralysis due to nervous injury.

The pressure in the glomeruli may also be diminished locally: 1. By section of the vaso-dilators. 2. By stimulation of the vaso-constrictors.

The composition of the blood affects secretion: 1. By dilution of the blood, causing excessive secretion, which may be caused by drinking a large quantity of water, or some specific disease. 2. Blood filled with effete material stopping the secretion.

Agents acting on the secretory centres may effect secretion: 1. By stimulating the secretory centre. 2. By stimulating the inhibitory secretory centres.

Mode of Action of Diuretics.—From what has been said it is evident that diuretics may act in several ways. They may act: 1. On the circulation in the kidney, raising the pressure in the glomeruli; (*a*) locally, by contracting the efferent vessels or by causing dilatation of the renal arteries, and thus increasing the supply of blood to the kidney; (*b*) generally, by causing the contraction of vessels in other parts of the body. 2. Other diuretics may act on the secreting cells of the tubules, and may increase both the amount of water and of solids excreted by them.

Diuretics may be classified as stimulative and sedative. The sedative class agrees very closely with the one we have just indicated as acting on the kidney through the circulation; (*a*) such as dilatation of the afferent vessels, by alcohol, nitritis, sweet spirit of nitre, which act either by stimulating the vaso-dilating nerves or paralyzing the vaso-constrictor nerves; (*b*) contraction of the efferent vessels by digitalis, strychnine, broom, buchu, juniper, turpentine, copaiba and cantharides, which are said to act by stimulating the ends of the vaso-constrictor nerves in the vessel wall. The ends of the secretory nerves in the cells may be stimulated by urea, caffeine, calomel, potassium acetate and other saline diuretics.

From what we said of the action of diuretics it will readily be admitted that we may hope to do much more by combining them than by using them singly.

Thus we may see that digitalis in large doses, instead of acting as a diuretic, may completely arrest the circulation and stop secretion. If, however, we combine it with some drug which will produce dilatation of the renal vessels, *e. g.*, sweet spirit of nitre, while the general blood pressure remains high, we will greatly increase the circulation through the kidney and have the desired effect.

